# Genetic diversity and population structure of *Cinnamomum balansae* Lecomte inferred by microsatellites

**DOI:** 10.1515/biol-2022-0037

**Published:** 2022-04-05

**Authors:** Bei Cui, Dinh Duy Vu, Dinh Giap Vu, Thi Tuyet Xuan Bui, Siddiq Ur Rahman, Mai Phuong Pham, Minh Tam Nguyen, Van Sinh Nguyen, Syed Noor Muhammad Shah, Viet Ha Tran

**Affiliations:** Key Comprehensive Laboratory of Forestry, College of Forestry, Northwest A & F University, Yang Ling 712100, China; Graduate University of Science and Technology (GUST), Vietnam Academy of Science and Technology (VAST), 18 Hoang Quoc Viet, Cau Giay, Hanoi, Vietnam; Department of Chemical Technology – Environment, HaUI Institute of Technology, Hanoi University of Industry (HaUI), 298 Cau Dien, Bac Tu Liem, Hanoi, Vietnam; Department of Plant Ecology, Institute of Ecology and Biological Resource, VAST, 18 Hoang Quoc Viet, Cau Giay, Hanoi, Vietnam; Department of Computer Science & Bioinformatics, Khushal Khan Khattak University, Karak, Khyber Pakhtunkhwa, 27200, Pakistan; Institute of Tropical Ecology, Vietnam – Russia Tropical Centre, 63 Nguyen Van Huyen, Cau Giay, Hanoi, Vietnam; Department of Experimental Taxonomy and Genetic Diversity, Vietnam National Museum of Nature, VAST, 18 Hoang Quoc Viet, Cau Giay, Hanoi, Vietnam; Graduate University of Science and Technology, VAST, 18 Hoang Quoc Viet, Cau Giay, Hanoi, Vietnam; Department of Horticulture, Faculty of Agriculture, Gomal University, Dera Ismail Khan, 29220, Pakistan; Faculty of Silviculture, Vietnam National University of Forestry, Xuan Mai, Chuong My, Hanoi, Vietnam

**Keywords:** bottleneck, *C. balansae*, conservation, genetic variability, fragmentation

## Abstract

*Cinnamomum balansae* Lecomte (Lauraceae), an economically important forest tree, is distributed in the tropical forests of central and northern Vietnam, which has been threatened in recent decades due to the destruction of its habitat and over-exploitation. The genetic diversity and population structure of the species have not been fully evaluated. We used a set of 15 microsatellites to analyze 161 adult trees from 9 different populations, representing the geographical distribution of *C. balansae*. Ninety-two different alleles were identified. Here our results showed a low genetic diversity level with an average *H*_o_ = 0.246 and *H*_e_ = 0.262, and a high level of genetic differentiation (*F*_ST_ = 0.601). The bottleneck tests indicated evidence of a reduction in the population size of the two populations (TC and CP). Additionally, all three clustering methods (Bayesian analysis, principal coordinate analysis, and Neighbor-joining tree) were identified in the two genetic groups. The Mantel test showed a significant positive correlation between genetic distance and geographic distance (*R* ^2^ = 0.7331). This study will provide a platform for the conservation of *C. balansae* both in *ex-situ* and *in-situ* plans.

## 1 Introduction

*Cinnamomum balansae* Lecomte, a large and evergreen tree of the Lauraceae family, is distributed in the tropical forests of central and northern Vietnam [1,2,3,4]. It is found at an elevation above 200 m in secondary forests on the ancient alluvial rocks and granite that have low relief and gentle slopes. The species prefers precipitation of 800–2,500 mm and an annual mean temperature of 20–22°C [2,4]. *C. balansae* can reach a height of 30 m with a diameter of 85–90 cm at breast height. Flowers are small, greenish-yellow, and appear between March and May. Fruiting appears from April to July, with fruit and maturation occurring in October [2]. Its bark has oil and camphor. The fruit oil and fat are used to make soap. Wood is used for furniture. In the past few decades, this species has been over-exploited by local people and forestry enterprises [1]. Its habitats have been destroyed and degraded [5,6]. For that reason, *C. balansae* is listed as an endangered species in the IUCN Red List [7] and the Vietnam Red Data Book [1].

The adaptive and evolutionary potential of a species depends on its genetic diversity [8]. Conservation and management of a species require knowledge of its ecological characteristics and genetic variation within and between populations [9,10,11]. To obtain such information, particularly a better understanding of genetics, powerful and biological techniques are required [12,13]. Molecular markers such as allozyme [14], randomly amplified polymorphic DNA (RAPD) [15,16], and chloroplastic DNA [17] have been used to investigate the genetic diversity of species in the Lauraceae family. Microsatellites (SSRs) are widely distributed throughout the nuclear genome, displaying high polymorphism and reproducibility [18,19,20]. These markers have been widely used to study the Lauraceae species [21,22,23,24,25,26,27,28,29]. In the current study, microsatellites were used to assess the genetic diversity and population structure of *C. balansae* and provide a platform for effective conservation strategies of this species in Vietnam.

## 2 Materials and methods

### 2.1 Plant materials

With Illumina sequencing for the development of microsatellites, we collected three plant tissues, including roots, leaves, and stems of *C. balansae* (one individual) from a wild population (Ba Vi National Park, Hanoi city). All sample tissues were immediately frozen in liquid nitrogen and stored at –80°C until RNA extraction.

**Figure 1:**
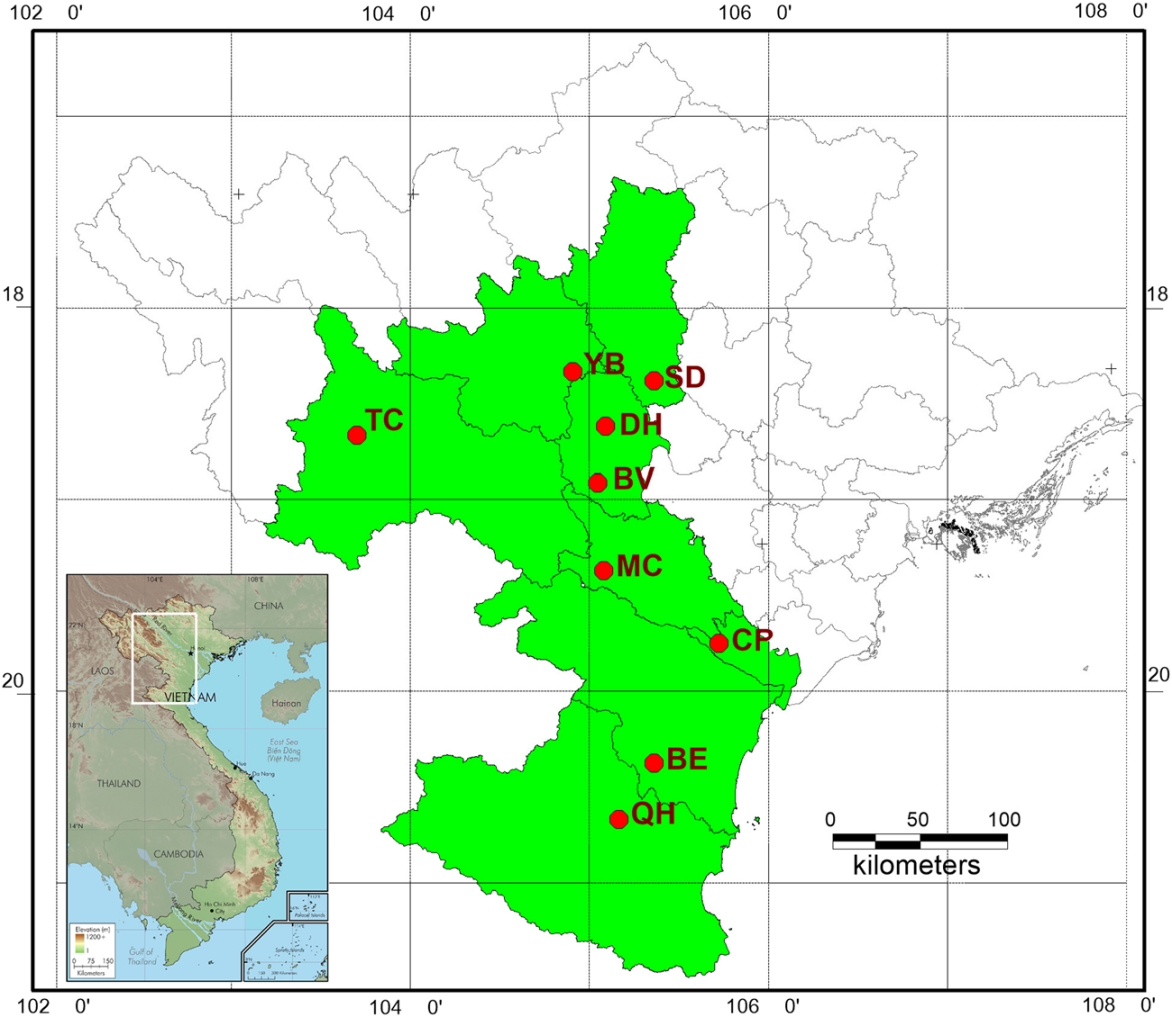
Map showing the collection sites of nine populations of *C. balansae* in Vietnam.

To assess the genetic variation in and population structure of *C. balansae*, the study area for this species was carried out in nine sites, one each in Hanoi, Phu Tho, Tuyen Quang, Yen Bai, Son La, Hoa Binh, Ninh Binh, Thanh Hoa, and Nghe An, representing its natural range in Vietnam (Table 1, Figure 1). Sampling locations were recorded using a global positioning system (GPS). The inner bark from 161 adult trees was randomly sampled for 9 known populations in central and northern Vietnam. Samples were placed into plastic bags containing silica gel in the field and then transferred to the Laboratory of Molecular Biology of Vietnam-Russia Tropical Centre and stored at −30°C until DNA extraction.

**Table 1:** Geographical characteristics of the nine *C. balansae* populations used in this study

Populations	Location	Sample size	Latitude (N)	Longitude (E)
BV	Ba Vi National Park, Ha Noi City (Northern Vietnam)	10	21°06′36″	105°33′16″
DH	Chan Mong commune, Doan Hung district, Phu Tho province (Northern Vietnam)	15	21°33′45″	105°09′49″
SD	Dong Bua commune, Son Duong district, Tuyen Quang province (Northern Vietnam)	19	22°10′15″	105°20′10″
YB	Van Lang Commune, Yen Binh district, Yen Bai province (Northern Vietnam)	12	21°41′52″	104°59′39″
TC	Chieng Bom Commune, Thuan Chau district, Son La province (Northern Vietnam)	16	21°38′87″	103°53′52″
MC	Tan Mai commune, Mai Chau district, Hoa Binh province (Northern Vietnam)	20	20°44′46″	104°55′32″
CP	Cuc Phuong National Park, Ninh Binh province (Central Vietnam)	21	20°21′06″	105°35′57″
BE	Ben En National Park, Thanh Hoa province (Central Vietnam)	21	19°58′08″	105°45′57″
QH	Tam Hop Commune, Quy Hop district, Nghe An province (Central Vietnam)	27	19°21′21″	105°17′05″

### 2.2 DNA/RNA extraction, cDNA sequencing, and microsatellite loci identification

For Illumina sequencing, total RNA from each sample was isolated using the OmniPlant RNA Kit (DNase I) (www.cwbiotech.com). Both quantity and quality of total RNA were checked through Nanodrop ND-2000 spectrophotometer (Thermo Electron Corporation, USA) and Agilent 2100 Bioanalyzer. An equal amount of total RNA from each sample was pooled together and sent to Breeding Biotechnologies Co. Ltd for cDNA library construction and transcriptome sequencing using Illumina HiSeq™4000. Cleaned mRNA was used for cDNA library construction extracted from 200 µg total RNA using Oligo (dT). The cDNA first strand was prepared from random hexamers using mRNA as a template, and the other strands were from the buffer, dNTPs, RNase H, and DNA polymerase I, and then cleaned with AMPure XP beads. The cleaned double-stranded cDNA was subjected to terminal repair, the sequencing linker was ligated, and then the fragment size was selected with AMPure XP beads. The cDNA library was acquired by PCR enrichment. After validating the library on an Agilent 2100 Bioanalyzer, Breeding Biotechnologies Co., Ltd sequenced the cDNA libraries on an Illumina HiSeq™ 4000. The Trimmomatic v3.0 [30] was used for raw reads filtration. The reads showing adaptor contamination, length < 36 bp, and low-quality value (quality <20) higher than 15% were eliminated. Trinity [31] with default parameters were used for de novo assembly of the cleaned reads. Then, the TIGR Gene Indices clustering tool (TGICL) v2.1 [32] was used to cluster and eradicate redundant transcripts and identified unigenes for further analysis.

SSRs were detected in the Simple Sequence Repeat Identification Tool program (SSRIT, http://www.gramene.org/db/markers/ssrtool). Only unigenes longer than 1 kb were included in the EST-SSR detection. The parameters were set to detect perfect, di-, tri-, tetra-, penta-, and hexa-nucleotide motifs with a minimum of 6, 5, 5, 5, and 5 repeats, respectively. Identification of microsatellite loci showed simple sequence repeats and was deposited in GenBank.

Total genomic DNA was extracted from the samples using the Plant Genome DNA Extraction Kit (BioTeke, Beijing, China). The quality of total DNA was verified by 1% agarose gel electrophoresis and NanoDrop2000 (BioTeke Instruments, Winooski, VT, USA). The concentration was then diluted to 20 ng/µL and stored at −20°C.

### 2.3 Microsatellite amplification

Polymerase chain reaction (PCR) was performed in a 25 μL of reaction mixture containing 2.5 µL of template DNA, 12.5 µL of 2X Taq Master mix, 1 µL of each primer, and 8 µL of deionized water. All SSR primers were designed using Primer 5.0 software [33]. The main stated selection criteria for the screening of primers were as follows GC content of 45–55 with 55% as the optimum; primer length of 18–24 bp with 20 bp as the optimum length; annealing temperature from 55 to 65°C, with 60°C as the optimum temperature; and PCR product size ranging from 100 to 300 bp. Fifteen SSR polymorphic markers were used in the present study (Table 2). The PCR amplification was carried out on a Mastercycler X50s Eppendorf, under the following thermal cycler: initial denaturation at 94°C for 2 min, followed by 40 cycles consisting of 1 min at 94°C for denaturation, 30 s alignment at the annealing temperature for each primer pair at 55°C and 1 min at 72°C for extension, and 10 min at 72°C for final cycle to complete the extension of any remaining products before holding the samples at 4°C until they were analyzed. The amplification products were separated on 8% polyacrylamide gels in 1 × TAE buffer using the Cleaver Scientific electrophoresis system (UK) and visualized with GelRed^TM^ 10000X. The sizes of the PCR products were detected and analyzed using GelAnalyzer software with a 20 bp DNA ladder (Invitrogen).

**Table 2:** Characteristics and genetic estimates for 15 SSR loci of *C. balansae*

Locus	Primer sequence (5′–3′)	Motif	Size (bp)	*N*_a_	*N*_p_	*F*_IS_	*F*_IT_	*F*_ST_	*N*_m_	GenBank code
V03	F: CCACCCCAATTCGATTATTG	(AGCAGG)_5_(AGC)_6_	219	5	2	0.335	0.645	0.467	0.285	MN486375
	R: CCAAGCAGCTTTACTCCACC									
V07	F: GAGAGCTTCCAAAGCCACTG	(CAC)_5_(CAT)_5_	234	6	3	0.635	0.864	0.626	0.149	MN486379
	R: AAGAAGCCGACAAGCAAAAA									
V08	F: AGGGATTTTGGAGATTTTGC	(CTT)_5_(CCT)_7_	150	9	4	−0.130	0.239	0.327	0.515	MN486380
	R: GGACGCCAAAACCAAATGTA									
V11	F: TTTGAGGGAAATGGTTCCTG	(TAG)_5_(TAA)_6_	194	6	2	−0.096	0.453	0.501	0.249	MN486383
	R: TTGGGCCAAAACAAGAAGAC									
V25	F: ACGAGTTTCCACCGATTACG	(ATC)_8_	268	5	2	−0.024	0.992	0.992	0.002	MN486397
	R: ACTCCTTTCAGCACCGATTG									
V30	F: TTGTTAAAAACACCAACCCCA	(TCA)_8_	200	5	2	−0.113	0.524	0.572	0.187	MN486402
	R: CAGTGGGCCAAGTGTATCCT									
V31	F: ACGTGAATGTGAATGGGGTT	(AAC)_9_	189	5	1	0.219	0.851	0.809	0.059	MN486403
	R: TAGGCAAAGACTCCGAAGGA									
V33	F: ATTCGGGTCTCTCTCCCTGT	(ACCA)_6_	249	8	2	0.220	0.620	0.513	0.237	MN486405
	R: CTCTCTCGCTGTCTCTGCCT									
V35	F: TGGAGAACAACTTTGGGAGG	(GATG)_6_	119	7	2	−0.258	0.518	0.617	0.155	MN486407
	R: TGTTCCATGTTACAGATACAG									
V41	F: ATATGGTCCCAACTCCCTCC	(ACCCA)_5_	203	8	4	0.289	0.808	0.729	0.093	MN486413
	R: ACCGTCACCAGATCATCCAT									
V51	F: TCCCAACTGGACGAAGTTCT	(ATGACC)_7_	237	4	1	0.505	0.834	0.665	0.126	MN486423
	R: TTTGCTCGCTGTTATGATGC									
V75	F: ATCCTCCCAAGGACGCTTAT	(CCT)_7_	133	7	3	0.287	0.625	0.473	0.278	MN486447
	R: CCTTCAAGGAAAGAAGGGCT									
V81	F: CACCACCTTCTCCTTCCAAA	(CTC)_7_	234	4	2	−0.351	0.740	0.808	0.060	MN486453
	R: TTGTTGGGGTCTCCAAACTC									
V93	F: CGGACAATTTTCAGGGATGA	(GTC)_7_	166	6	2	−0.096	0.594	0.630	0.147	MN486465
	R: CCTGCATTCTAGATGCCTCC									
V108	F: GAAAGAAGTTGGGGGAGGAG	(TGA)_7_	209	6	3	−0.315	0.060	0.285	0.627	MN486480
	R: GAATCGCGGAGATACGGATA									
Mean				6.1		0.074	0.624	0.601	0.211	

*N*_a_: number of alleles; *N*_p_: private alleles; *F*_IS_: inbreeding coefficient; *F*_IT_: coefficient of total inbreeding; *F*_ST_: genetic differentiation; *N*_m_: gene flow.

### 2.4 Data analysis

Genetic diversity was estimated using GenAlEx [34] based on SSR allele frequencies, including the number of alleles per locus (*N*_a_), effective alleles (*N*_e_), private alleles (*N*_p_), the proportion of polymorphic loci (*P%*), the coefficient of total inbreeding (*F*_IT_), the genetic differentiation (*F*_ST_), Nei’s genetic distances among the populations, the observed heterozygosities (*H*_o_), and the expected heterozygosities (*H*_e_). Gene flow between populations (*N*_m_) was calculated using the *F*_ST_ value: *N*_m_ = (1/F_*ST*_ − 1)/4. The inbreeding coefficients (*F*_IS_) value was also calculated for each population by Fstat [35]. Tests for deviation from the Hardy-Weinberg equilibrium at each locus and the linkage disequilibrium for each locus pairwise combination in each population were also performed by Genpop v.4.6 [36]. Arlequin v.3.0 [37] was used to perform an analysis of molecular variance (AMOVA) for the estimation of genetic divergence as PhiPT, and its associated significance based on 10,000 permutations. Testing for recent bottleneck events for each population using Bottleneck v.1.2 [38] was applied with the stepwise mutation model (SMM) and two-phase model (TPM), and their significance was tested. The proportion of the SMM was set to 70% under default settings. The significance of these tests was evaluated by the one-tailed Wilcoxon signed-rank test.

Principal coordinate analysis (PCoA) was implemented based on *G*′_ST_ values [39] using GenAlEx. Neighbor-joining (NJ) tree was constructed genetic relationships among populations based on a matrix of *F*_ST_ values using Poptree2 [40]. Bayesian analysis of population structure was performed with Structure v.2.3.4 [41] to determine the optimal value of the genetic cluster (*K*). Setting the admixture model with correlated allele frequency and the ancestry models, 10 separate runs of the number of clusters (*K*) in the dataset were performed from 1 to 10 for each *K* value at 500,000 Markov Chain Monte Carlo (MCMC) repetitions and a 100,000 burn-in period. The optimal value of *K* was detected using Structure Harvester [42] based on the ∆*K* value by Evanno et al. [43].

A Mantel test [34] was applied to determine the relationships between genetic and geographic distances among the *C. balansae* populations based on 999 permutations.

## 3 Results

### 3.1 Genetic diversity

A total of 92 different alleles were detected from 15 SSR markers in *C. balansae* in the tropical forests of Vietnam. Allele numbers ranged from 4 (VD51 and VD81) to 9 (VD08), with an average of 6.1 (Table 2). Among these, 35 alleles were private. Loci VD08 and VD49 had the greatest number of private alleles (4). One private allele at 2 loci (VD31 and VD51), 3 private alleles at 3 loci (VD07, VD75, and VD108), and 2 private alleles at 8 remaining loci were recorded.

Genetic diversities for *C. balansae* populations are presented in Table 3. The average percentage of polymorphic loci was 68.15%, ranging from 60% in the 4 populations of BV, SL, TQ, and NA to 86.67% in the TH population. The average value of alleles (*N*_a_) per population was 1.9, ranging from 1.6 (SL) to 2.2 (YB and TH).

**Table 3:** Genetic diversity estimates nine populations of *C. balansae*

Population	*N*	*N*_a_	*N*_p_	*N*_e_	*P*%	*H*_o_	*H*_e_	*F*_IS_	*p* value of bottleneck	
									TPM	SMM
BV	10	1.7	2	1.5	60	0.267	0.275	0.003	0.049	ns
MC	20	1.8	4	1.4	73.33	0.260	0.253	−0.011	ns	ns
DH	15	1.7	4	1.4	66.67	0.219	0.213	−0.028**	ns	ns
TC	16	1.6	1	1.5	60	0.250	0.267	0.073	0.014	0.014
SD	19	1.7	1	1.4	60	0.193	0.228	0.101	ns	ns
YB	12	2.2	6	1.7	73.33	0.339	0.338	−0.051	0.042	ns
BE	21	2.2	6	1.4	86.67	0.270	0.253	−0.076	ns	ns
CP	21	1.9	8	1.7	73.33	0.270	0.334	0.212*	0.0003	0.0002
QH	27	1.9	6	1.4	60	0.143	0.198	0.179	ns	ns
Mean		1.9		1.5	68.15	0.246	0.262	0.025		

*N*: sample size; *p*: percentage of polymorphic loci; *N*_a_: alleles per locus; *N*_p_: total number of private alleles; *N*_e_: effective alleles; *H*_o_ and *H*_e_: observed and expected heterozygosities; *F*_IS_: inbreeding coefficient; *p < 0.05, **p < 0.01.

The highest number of private alleles (*N*_p_ = 8) was detected in the CP population, followed by three populations of YB, BE, and QH with *N*_p_ = 6, two populations of MC and DH with *N*_p_ = 4, the BV population (*N*_p_ = 2), and two populations (TC and SD) with *N*_p_ = 1. The *N*_e_ value varied from 1.4 in 5 different populations (MC, DH, SD, BE, and QH) to 1.7 (YB population), with an average of 1.5 alleles. The observed heterozygosity (*H*_o_) and expected heterozygosity (*H*_e_) averaged 0.246 (0.143–0.339) and 0.262 (0.198–0.338), respectively. The QH population had the lowest genetic diversity (*H*_o_ = 0.143 and *H*_e_ = 0.198) and the YB population had the highest values (*H*_o_ = 0.339 and *H*_e_ = 0.338). Five populations of BV, SD, TC, CP, and QH had positive *F*_IS_ values, indicating an excess of homozygosity and inbreeding in these populations. The highest *F*_IS_ value (0.212) was found in the CP population and was significant (*p* < 0.05). A significant deficit of heterozygosity was detected in two populations (TC and CP), based on the Bottleneck analysis under the TPM and SMM (*p* < 0.05), and suggested evidence of a bottleneck.

### 3.2 Population structure and Mantel test

The average *F*_IT_ value was 0.624, ranging from 0.06 (VD108) to 0.992 (VD25), indicating a deficiency of heterozygosity in the populations (Table 2). The genetic differentiation among populations (*F*_ST_) varied from 0.285 (VD108) to 0.992 (VD25) with an average of 0.601, indicating high differentiation among all the populations and reflecting low gene flow (*N*_m_ = 0.211). Our results of the AMOVA showed that the proportion of genetic variation among the populations was higher (74%) than within populations (Table 4). The PhiPT value and gene flow among populations were 0.744 and 0.086, respectively. The PhiPT value for the variation among populations was significant (*p* < 0.001).

**Table 4:** Analysis of molecular variance from nine populations of *C. balansae*

	df*	Sum of squares	Variance components	Proportion of variation (%)	PhiPT value	*N*_m_
Among populations	8	1307.8	13.592	74	0.744**	0.086
Within populations	102	477.5	4.681	26		
Total	110	1785.3	18.273			

df* degree of freedom; **p < 0.001.

**Figure 2:**
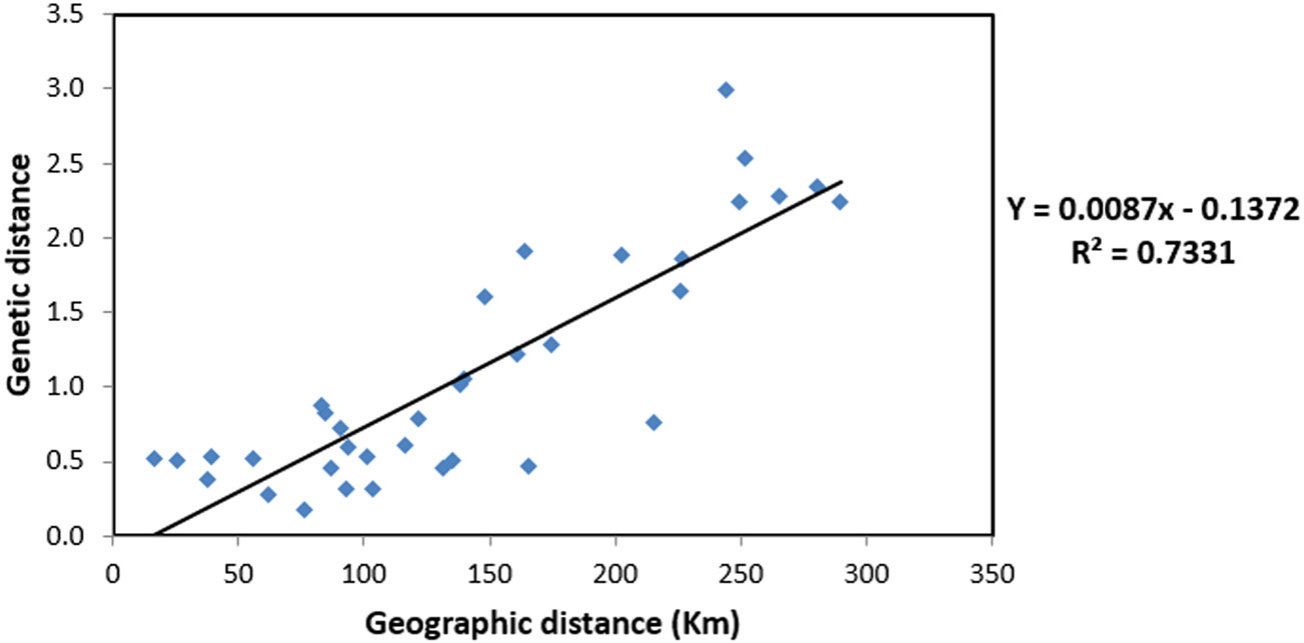
Mantel test of genetic distance and geographical distance of nine *C. balansae* populations.

The Mantel test revealed a significantly high correlation between genetic distance and geographical distance (*R* ^2^ = 0.7331) (Figure 2), indicating high genetic differentiation among populations.

**Figure 3:**
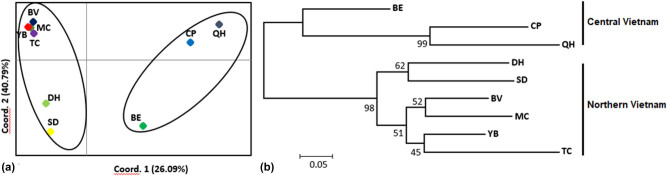
Principal Coordinates (PCoA) (a) and NJ tree for nine populations of *C. balansae* with bootstrap values above branches (b).

**Figure 4:**
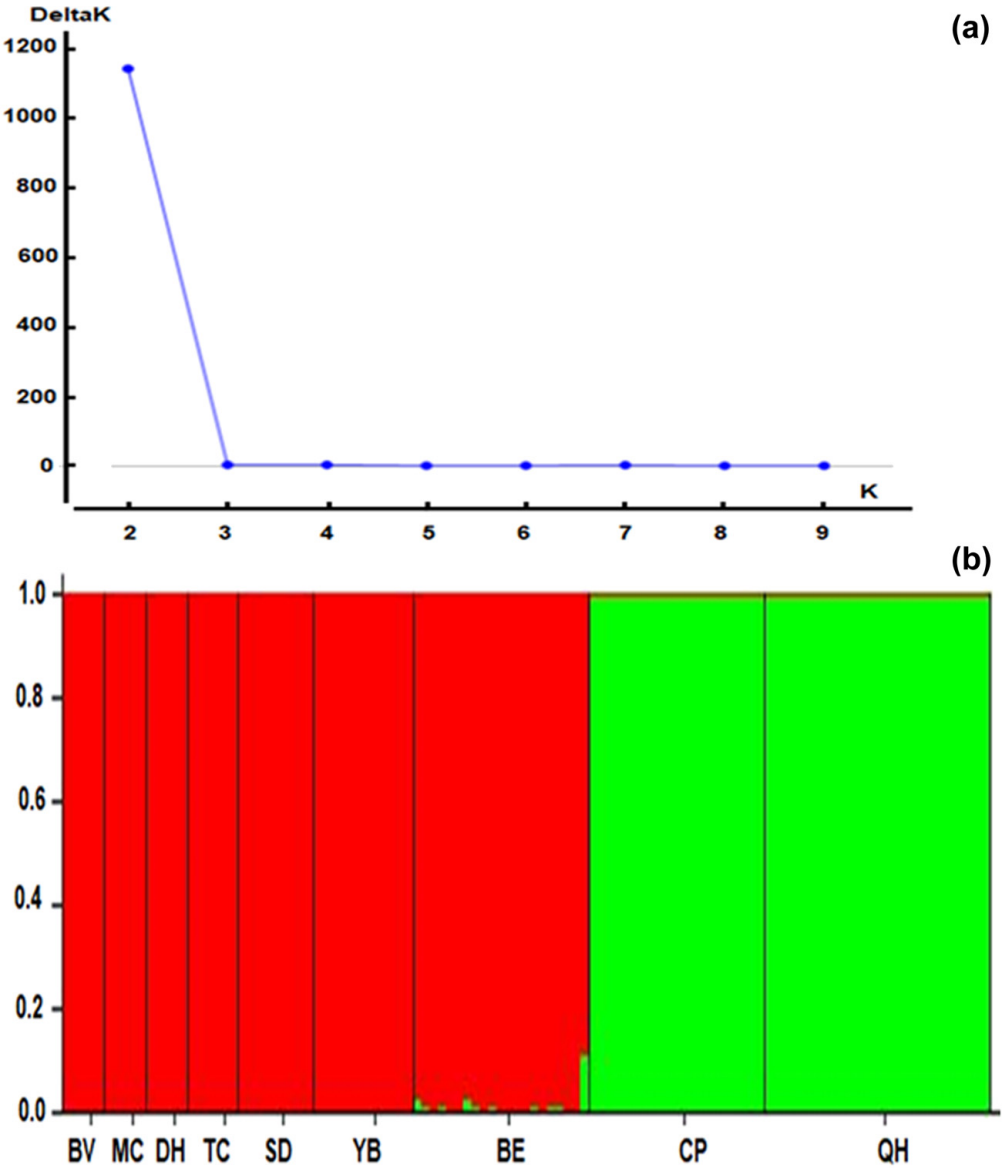
The Delta *K* distribution graph (a) and bar plot of admixture assignment for nine *C. balansae* populations to cluster (*K* = 2 and highest ∆*K* value = 1145.4) based on Bayesian analysis (b).

Genetic clusters were distinguished through the NJ analysis by using Poptree2 and the PCoA based on *G*′_ST_ values (Figure 3). Two different clusters were generated. The first cluster included three populations (CP, BE, and QH) in the central region, while the second included the remaining six populations (DH, SD, BV, MC, YB, and TC) in the northern region. An admixture model was performed to evaluate the population structure of the 161 individuals of *C. balansae* based on the Bayesian analysis using the Structure program. The results showed that the optimum number of genetic groups (*K*) was two, with the highest value of delta *K* (1,145.4) obtained from Structure Harvester might an optimum number of genetic clusters (Figure 4). Each individual in each population was assigned to one of the two genetic clusters. The ancestry coefficients of each individual and each population are shown in Figure 4. The red cluster represented all individuals from the populations of BV, MC, DH, TC, SD, and YB, while the green cluster represented CP and QH populations. All the individuals in the BE population were separated and closer to the red cluster than the green cluster.

## 4 Discussion

The genetic diversity of a species is often correlated with its history and ecology [44,45,46]. The genetic diversity is positively correlated with geographic distribution range and population size [47]. Species with widespread distribution, large population size, longevity, and outcrosses often maintain high genetic diversity compared to species with a narrow distribution [48]. Here our results indicated that *C. balansae* has low genetic diversity (*H*_o_ = 0.246 and *H*_e_ = 0.262) and high genetic differentiation among populations (*F*_ST_ = 0.601), compared to other widespread species, such as *C. camphora* in South China (*H*_o_ = 0.45 and *H*_e_ = 0.44) [29], *Albanian olive* (*H*_o_ = 0.75 and *H*_e_ = 0.60) [49 and E*ugenia dysenterica* (*H*_o_ = 0.545 and *H*_e_ = 0.62) [50]. Although this species has a widespread distribution and long life, the decline in genetic diversity may be related to anthropogenic disturbances, such as deforestation, habitat degradation, and over-exploitation of *C. balansae* [51]. Furthermore, in the current study, positive *F*_IS_ values were observed in the five populations of BV, TC, SD, CP, and QH, while the negative *F*_IS_ values were found in the remaining four populations of MC, DH, YB, and BE. A heterozygosity deficit was determined by the Bottleneck analysis, which detected a reduction in the population size of *C. balansae*. Small populations might be the result of inbreeding, which can decrease genetic variation. However, the bottlenecks also show a rapid reduction in the population sizes of TC and CP in recent decades. A high level of genetic differentiation (*F*_ST_ = 0.601) was determined, and reflected low gene flow (*N*_m_ = 0.211) among *C. balansae* populations. This is in contrast to previous reports on widespread species, such as *Dalbergia cochinchinensis* (*F*_ST_ = 0.23) [52] and *Erythrophleum fordii* Oliv (*F*_ST_ = 0.18) [53]. The dispersal of pollen and seeds plays an important role in maintaining high genetic diversity within and low genetic differentiation among populations [54,55]. The large geographic distance between *C. balansae* populations may be responsible for high genetic differentiation. Moreover, habitat fragmentation can increase the isolation between populations. Thus, these suggested the geographic distance and habitat fragmentation as a barrier to restrict gene flow between populations. Population structure and genetic relationships are important for establishing the appropriate scale and subunits for species conservation and management [56]. The study also identified two genetic groups using NJ, Structure, and PCoA: one in northern Vietnam (DH, SD, BV, MC, YB, and TC populations), and the other in central Vietnam (BE, CP, and QH populations). The Mantel test shows that there are almost obvious geographical boundaries between the studied populations, and it indicated the effects of the geographic distance on the number of migrants per generation inferred from the studied loci. This suggests that past human activities can have a significant impact on the present population structure and genetic diversity of *C. balansae* in Vietnam. Thus, the best conservation strategy should be to establish an *ex-situ* conservation site for this species through collecting seeds, germinating, and cultivating seedlings. Collecting seeds from individuals of all the populations should be conducted so that random loss of genetic variability due to loss of individual ecotypes should be avoided and move towards new self-sustaining populations. The establishment of a new large population of *C. balansae* should provide new alleles, which might improve its fitness under different environmental stresses to prevent the gradual reduction in the population of *C. balansae*.

## 5 Conclusion

These results confirmed that *C. balansae* had low genetic diversity and high genetic differentiation among populations in the evergreen tropical forests. The nine populations of *C. balansae* were divided into two genetic groups related to the geographic distance. Our study will contribute to understanding the population history of this species and the genetic relationships among the *C. balansae* populations.
